# OUtcome and Clinical Characteristics of Primary Headache in Patients with Sarcoidosis: The OUCH! Study

**DOI:** 10.3390/life16050762

**Published:** 2026-05-02

**Authors:** Claudio Tana, Nicol Bernardinello, Giacomo Giulianelli, Samanta Moffa, Francesco Cinetto, Laura Martino, Lucia Buzzelli, Maria Adele Giamberardino, Francesco Cipollone, Filippo Martone, Marco Tana, Livia Moffa, Paolo Spagnolo

**Affiliations:** 1 Internal Medicine Unit, Eastern Hospital, ASL Taranto, 74024 Manduria, Italy; 2 Respiratory Disease Unit, Department of Cardiac, Thoracic, Vascular Sciences and Public Health, University of Padova, 35122 Padova, Italy; nicol.bernardinello@unipd.it (N.B.); giacomo.giulianelli@studenti.unipd.it (G.G.); paolo.spagnolo@unipd.it (P.S.); 3 Department of Pharmacy, G. D’Annunzio University of Chieti, 66100 Chieti, Italy; samanta.moffa@unich.it; 4 Rare Diseases Referral Center, Internal Medicine 1, Ca’ Foncello Hospital, University of Padova, 31100 Padova, Italy; francesco.cine@gmail.com; 5 Respiratory Disease Unit, ASL2 Lanciano Vasto Chieti, 66034 Chieti, Italy; laura.martino@asl2abruzzo.it; 6 Headache Center, ASL2 Lanciano Vasto Chieti, 66100 Chieti, Italy; lucia.buzzelli@studenti.unich.it (L.B.); mag@unich.it (M.A.G.); fcipollone@unich.it (F.C.); 7 Amici Contro la Sarcoidosi Italia (ACSI), 40137 Bologna, Italy; presidenza@sarcoidosi.org; 8 Internal Medicine Unit, ASL2 Lanciano Vasto Chieti, 66100 Chieti, Italy; marco_tana@yahoo.it; 9 Infectious Disease Clinic and Internal Medicine Department, G. D’Annunzio University of Chieti, ASL2 Lanciano Vasto Chieti, 66100 Chieti, Italy

**Keywords:** pain, migraine, sarcoidosis, comorbidity, inflammation

## Abstract

Background: Headache is a frequent but often underestimated complaint in patients with sarcoidosis. In clinical practice, headache is commonly interpreted as secondary to neurosarcoidosis, potentially overlooking the presence of primary headache disorders, particularly migraine. The prevalence and clinical relevance of migraine in sarcoidosis remain insufficiently characterized. Objective: To investigate the prevalence and clinical characteristics of migraine in patients with sarcoidosis and to explore its association with pulmonary functional outcomes. Methods: The OUtcome and Clinical characteristics of primary Headache in patients with Sarcoidosis (OUCH!) Study is a multicenter, retrospective, observational study including adult patients evaluated at pulmonology outpatient clinics and headache centers between January 2019 and January 2021. Demographic, clinical, radiological, and pulmonary function data were collected. Patients were stratified according to the presence or absence of migraine. Pulmonary function parameters were compared using non-parametric statistical tests. Results: Seventy-two patients with sarcoidosis were included; 21 (29.2%) were diagnosed with migraine. Migraine prevalence was higher than expected for the general population. Pulmonary involvement was the most frequent disease manifestation. Patients with migraine showed significantly lower DLCO values compared with those without migraine (median (IQR): 55 (40–70) vs. 78 (65–90); *p* = 0.0009). No significant differences were observed in spirometric parameters or radiological patterns between groups. Conclusions: Migraine is a common comorbidity in sarcoidosis and is associated with reduced DLCO, suggesting a link with greater functional disease burden rather than structural lung damage. Migraine should be recognized as a primary headache disorder in this population, rather than automatically attributed to neurosarcoidosis. These findings support a multidisciplinary, patient-centered approach and warrant prospective studies to clarify shared inflammatory, vascular, and neuroimmune mechanisms.

## 1. Introduction

Sarcoidosis is a complex multisystem inflammatory disease characterized by the presence of non-caseating granulomas at disease sites and an unpredictable clinical course [1]. Non-caseating granulomas are organized collections of inflammatory cells lacking central necrosis, in contrast to granulomas seen in infectious conditions such as tuberculosis [1]. Although pulmonary and intrathoracic lymph node involvement represent its most common and recognizable features, sarcoidosis frequently extends beyond the respiratory system, affecting multiple organs and resulting in a wide spectrum of clinical presentations [2]. This intrinsic heterogeneity often complicates diagnosis, disease stratification, and long-term management, particularly when symptoms are subtle, nonspecific, or overlap with common clinical conditions [1,2,3].

Among the less clearly defined manifestations of sarcoidosis, headache represents a frequent but underestimated complaint [4]. In routine clinical practice, headache in patients with sarcoidosis is commonly interpreted as a secondary phenomenon, attributed to neurosarcoidosis, meningeal inflammation, intracranial hypertension, or structural central nervous system involvement [4]. While these conditions must be carefully excluded, such an approach risks overlooking a substantial proportion of patients in whom headache fulfills criteria for a primary headache disorder, most notably migraine [5]. This distinction is clinically relevant, as primary and secondary headaches differ substantially in terms of pathophysiology, prognosis, and therapeutic strategies [6].

Migraine is a prevalent and disabling primary headache disorder that is increasingly recognized as a systemic condition rather than a purely neurovascular disease [6]. Immune activation, cytokine signaling, and neuroinflammatory mechanisms are believed to play a central role in disease pathophysiology [6]. These processes conceptually intersect with the immune dysregulation underlying sarcoidosis, suggesting a potential biological substrate for their coexistence. Nevertheless, the clinical implications of this overlap remain poorly characterized [7].

To date, only a limited number of studies have specifically investigated primary headache disorders in patients with sarcoidosis. Available evidence suggests that migraine may be more prevalent in this population than in the general population, with female sex emerging as a potential risk factor [8]. However, existing studies are often limited by their small sample size, incomplete phenotypic characterization, and lack of outcome-focused analyses. In particular, it remains unclear whether the presence of migraine identifies a distinct sarcoidosis phenotype or influences disease severity, pulmonary function, or broader clinical outcomes [8].

Understanding the burden of primary headache disorders in sarcoidosis is essential to fully appreciate the impact they could have on patient care and quality of life. Headache, and migraine in particular, may substantially contribute to symptom burden, complicate clinical assessment, and influence therapeutic decision-making [7]. Failure to recognize primary headache disorders in this context may lead to misdiagnosis, unnecessary investigations, or suboptimal treatment strategies. A more precise characterization of headache phenotypes in sarcoidosis could therefore support a more integrated and patient-centered approach, supporting the collaboration between pulmonologists, neurologists, and headache specialists [7,8].

The OUtcome and Clinical characteristics of primary Headache in patients with Sarcoidosis (OUCH!) Study was designed to address these gaps. By investigating the prevalence and clinical characteristics of migraine in a real-world cohort of patients with sarcoidosis, and by comparing clinical and pulmonary outcomes between patients with and without migraine, this study aims to provide a clearer framework for understanding the role of primary headache disorders within the complex clinical landscape of sarcoidosis.

## 2. Methods

### 2.1. Study Design

The OUCH! Study is a multicenter, retrospective, observational, non-profit study conducted in patients with a confirmed diagnosis of sarcoidosis and/or migraine. Patients were evaluated at dedicated Pulmonology outpatient clinics and Headache Centers of the participating institutions.

### 2.2. Study Population and Inclusion/Exclusion Criteria

Adult patients (≥18 years) of both sexes with a documented diagnosis of sarcoidosis and/or migraine who were evaluated between January 2019 and January 2021 at participating Pulmonology or Headache outpatient clinics were eligible for inclusion. Inclusion criteria were age ≥ 18 years, a confirmed diagnosis of sarcoidosis and/or migraine, and clinical evaluation performed at the participating centers. Patients with active malignancy, either pulmonary or extrapulmonary, were excluded from the study. Sarcoidosis diagnosis was established according to established clinical, radiological, and histological criteria, while migraine diagnosis was established according to the International Classification of Headache Disorders, 3rd edition (ICHD-3) criteria.

### 2.3. Data Collection

Clinical data were retrospectively retrieved from medical records and included demographics, medical history (personal and family history, cardiovascular risk factors, smoking habits, and environmental exposures), clinical characteristics of migraine and sarcoidosis, physical examination findings (blood pressure, heart rate, oxygen saturation, and body mass index), pulmonary function tests including spirometry with forced expiratory volume in one second (FEV_1_), forced vital capacity (FVC), diffusing capacity of the lung for carbon monoxide (DLCO), and total lung capacity (TLC), chest computed tomography features, and ongoing therapies, including disease-specific and home treatments. Laboratory parameters were collected when available and are reported in Supplementary Table S1.

All data were anonymized and recorded in a dedicated electronic case report form.

### 2.4. Histological Findings and Therapy

Histological findings were reclassified into standardized diagnostic categories: histologically confirmed sarcoidosis (presence of non-necrotizing granulomas), compatible granulomatous pattern, non-diagnostic or nonspecific biopsy, and biopsy not performed. Furthermore, the type of treatment administered to patients was reported.

### 2.5. Study Outcomes

The primary objective was to investigate the clinical characteristics of patients with coexisting migraine and sarcoidosis compared with patients with migraine alone.

The secondary objective was to evaluate clinical outcomes of patients with sarcoidosis with and without migraine, including pulmonary function impairment, chest CT findings, hospitalizations, and mortality.

### 2.6. Statistical Analysis

Patients were divided into two groups based on the presence of migraine. Respiratory function parameters, including FEV_1_, DLCO, and TLC, were compared between the two groups using the Mann–Whitney U test, as data were not normally distributed, as assessed by the Shapiro–Wilk test. A multivariate analysis was additionally conducted to evaluate the association between migraine and DLCO, adjusting for potential confounders including age, sex, family history, pleuro-pulmonary diseases, BMI, dyslipidemia, hypertension, diabetes, and atherosclerosis. Variables with substantial missing data were not included. Results were considered statistically significant at *p* values < 0.05. All statistical analyses were performed using GraphPad Software v.8, Inc. (San Diego, CA, USA).

### 2.7. Ethical Considerations

The study was conducted in accordance with the Declaration of Helsinki and the principles of Good Clinical Practice. The study protocol was approved by the Ethics Committee of the coordinating center and subsequently approved by the Ethics Committees of the participating satellite centers (protocol number of the coordinating Ethics Committee: Prot. No. 0445105/23; approval date: 12 February 2024).

Given the retrospective and observational design, clinical data were collected from medical records and processed in anonymized form, in compliance with the EU General Data Protection Regulation (GDPR 2016/679) and with the Italian Data Protection Authority authorization No. 9/2016, including its subsequent extension (Provision No. 424/2018).

Written informed consent for study participation and data processing was obtained from all patients, in accordance with local ethical and regulatory requirements.

No financial compensation was provided to investigators or participants, in line with regulations governing non-profit observational studies.

## 3. Results

### 3.1. Study Population

A total of 72 patients were included in the analysis, comprising 33 females and 39 males (Table 1). All patients had a confirmed diagnosis of sarcoidosis, with or without concomitant primary headache disorders. Among the study population, 21 patients (29.2%) were diagnosed with migraine, whereas 51 patients (70.8%) did not report migraine.

### 3.2. Sarcoidosis Clinical Patterns

Pulmonary involvement represented the predominant clinical manifestation. The most frequent disease patterns were:

- Parenchymal and mediastinal sarcoidosis in 25 patients (34.72%);
- Parenchymal sarcoidosis alone in 9 patients (12.5%);
- Isolated mediastinal lymph node involvement or minimal parenchymal disease in 8 patients (11.12%);
- Mediastinal and pulmonary micronodules or opacities in 2 patients (2.78%);
- Pulmonary fibrosis in 1 patient (1.38%);
- Combined pulmonary and cardiac sarcoidosis in 1 patient (1.38%);
- Sarcoidosis with unspecified involvement in 26 patients (36.12%).

Overall, the lung was the most common disease site in this cohort. These patterns reflect the distribution of organ involvement observed in the study cohort rather than predefined categories, although they are consistent with clinical manifestations commonly described in the literature.

### 3.3. Risk Factors and Family History

Most patients (39) reported no identifiable risk factors. Twenty-seven patients reported exposure to smoking and/or air pollution, while risk factor data were unavailable for 6 patients. Family history was largely negative, with only isolated cases reporting autoimmune or thrombotic conditions in first-degree relatives.

### 3.4. Comorbidities

The prevalence of major comorbidities was relatively low. Hypertension was present in 22 patients, dyslipidemia in 9, diabetes mellitus in 4, and atherosclerotic disease in 5 patients. The majority of patients did not present significant cardiovascular or metabolic comorbidities. Body mass index (BMI) distribution revealed normal weight in 22 patients, overweight in 19, obesity in 12, and underweight in one patient; BMI data were missing in 18 patients.

### 3.5. Pulmonary Function Tests

Spirometry was abnormal in 28 patients and normal in 32, whereas data were unavailable for 12 patients.

Regarding airflow limitation, FEV_1_ ≥ 80% of predicted was observed in 32 patients, while mild to moderate reductions (50–79%) were found in 15 patients. Severe or very severe impairment (FEV_1_ < 50%) was documented in 13 patients. FEV_1_ data were not reported in 15 patients.

Diffusing capacity for carbon monoxide (DLCO) was normal in 21 patients, mildly reduced in 25, and moderately to severely reduced in 10 patients; DLCO data were unavailable for 16 patients.

Overall, pulmonary function impairment was heterogeneous, with a substantial proportion of patients showing clinically relevant functional involvement.

**Figure 1:**
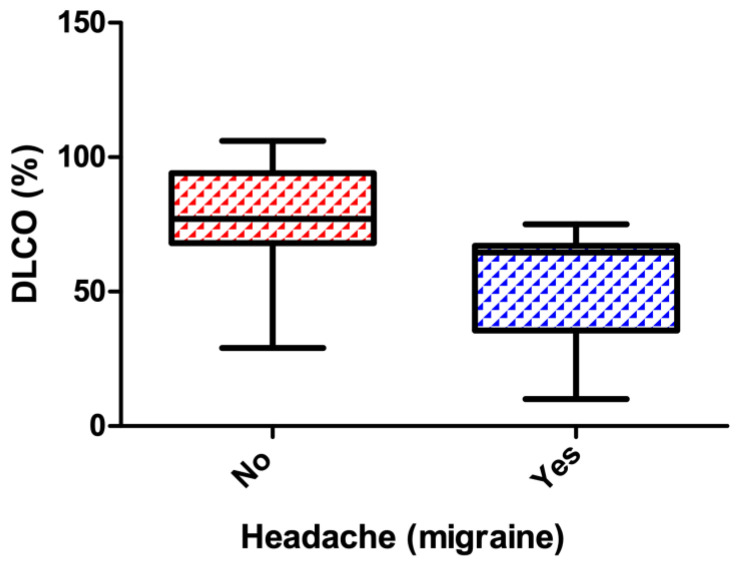
Association between DLCO levels (%) and presence or absence of migraine in patients with sarcoidosis (*p* value < 0.001).

DLCO levels were significantly lower in patients with migraine (54.91 ± 20.74) compared with those without migraine (77.93 ± 18.92), with median [IQR] values of 55 [40–70] and 78 [65–90], respectively (*p* = 0.0009) (Figure 1).

### 3.6. Multivariable Analysis

To assess whether the association between migraine and DLCO was independent of potential confounders, a multivariable linear regression model was constructed including age, sex, BMI, and major comorbidities (Table 2).

**Figure 2:**
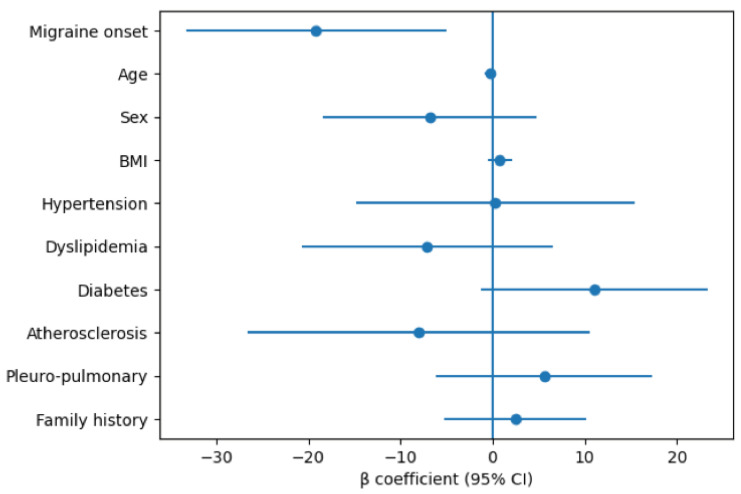
Multivariable regression analysis for DLCO. Forest plot showing β coefficients and 95% confidence intervals for variables included in the multivariable linear regression model. Migraine onset was significantly associated with lower DLCO values, whereas all other covariates showed non-significant associations, with confidence intervals crossing zero.

In the adjusted model, migraine onset remained significantly associated with lower DLCO values (β = −19.15; 95% CI: −33.28 to −5.02). None of the other covariates showed a statistically significant association with DLCO (Figure 2). However, the overall model did not reach statistical significance (F(10,44) = 1.50; *p* = 0.17) and explained approximately 25% of the variability in DLCO values (R^2^ = 0.25).

## 4. Discussion

In this multicenter real-world study, migraine emerged as a frequent comorbidity in patients with sarcoidosis, affecting nearly one-third of the cohort. This prevalence is higher than that reported in the general population and is consistent with previous observations suggesting an increased burden of primary headache disorders in sarcoidosis [8]. These findings support the notion that headache in sarcoidosis should not be automatically interpreted as a secondary manifestation or as a marker of central nervous system involvement, but rather warrants careful clinical phenotyping [9].

One of the most notable findings of the present study is the association between migraine and lower DLCO values in patients with sarcoidosis. While these two conditions represent distinct clinical and pathophysiological entities, the observed relationship suggests that migraine may cluster with a subgroup of patients characterized by greater functional impairment. DLCO is a sensitive indicator of alveolar–capillary dysfunction and pulmonary vascular involvement in sarcoidosis, and its reduction has been associated with disease severity and prognosis [10,11]. Importantly, this finding should be interpreted with caution, as it does not imply a causal relationship between migraine and pulmonary functional impairment, but rather highlights a potential shared or parallel vulnerability.

Several hypothesis-generating mechanisms may help explain this association. First, systemic inflammation and immune dysregulation, which are central features of sarcoidosis, may contribute to both impaired pulmonary diffusion and migraine susceptibility through overlapping inflammatory and endothelial pathways [7,12]. Cytokine-mediated endothelial activation and microvascular dysfunction have been implicated in migraine pathophysiology and could, in parallel, influence alveolar–capillary gas exchange [12]. Second, migraine may represent a clinical marker of a broader microvascular or neuroimmune phenotype, rather than a direct contributor to pulmonary dysfunction [13]. Third, autonomic imbalance and altered vascular reactivity, described in both migraine and systemic inflammatory diseases, could act as common modulators of these apparently unrelated clinical manifestations [12,13].

Notably, the association between migraine and DLCO was observed in the absence of clear differences in spirometric parameters or radiological disease patterns, suggesting that migraine may be linked to functional rather than structural aspects of pulmonary involvement. This further supports the hypothesis that subtle vascular or inflammatory mechanisms, rather than overt parenchymal damage, may underlie the observed findings [13].

From a clinical standpoint, these findings do not support interpreting migraine as a surrogate marker of neurosarcoidosis or as an indicator of direct central nervous system involvement. Rather, in most cases, migraine fulfills established diagnostic criteria for a primary headache disorder and should be recognized and managed accordingly [14]. This distinction is clinically relevant, as misattributing migraine symptoms to neurosarcoidosis may prompt unnecessary neuroimaging, invasive procedures, or escalation of immunosuppressive therapies, ultimately exposing patients to avoidable risks and contributing to inappropriate management strategies.

At the same time, the coexistence of migraine and sarcoidosis may substantially amplify the overall symptom burden and negatively impact quality of life. Migraine-related disability characterized by recurrent pain, photophobia, phonophobia, and functional impairment may overlap with or exacerbate key symptoms of sarcoidosis, including fatigue, chronic pain, and reduced physical functioning, which are themselves multifactorial and often difficult to quantify [15]. This overlap may complicate clinical assessment, leading to an overestimation of inflammatory disease activity or, conversely, to an under-recognition of treatable comorbid conditions.

Furthermore, migraine may influence treatment adherence and patient-reported outcomes, contributing to increased healthcare utilization and a greater psychosocial burden. In this context, recognizing migraine as a comorbidity rather than a direct complication of sarcoidosis is essential to ensure appropriate diagnostic pathways and tailored therapeutic interventions. Such an approach supports a more integrated, multidisciplinary, and patient-centered model of care, promoting collaboration between pulmonologists, neurologists, and headache specialists, and ultimately improving both clinical outcomes and quality of life.

### 4.1. Therapeutic Considerations and Clinical Implications

From a therapeutic perspective, the management strategies observed in our cohort reflect the marked heterogeneity of sarcoidosis in terms of clinical presentation, organ involvement, and disease course [16].

Systemic corticosteroids represented first-line therapy in most treated patients, with variable initial doses followed by gradual tapering and, in many cases, eventual discontinuation due to disease stabilization or adverse effects. In patients with chronic, relapsing, or steroid-dependent disease, immunosuppressive and steroid-sparing agents, most commonly methotrexate, but also azathioprine, mycophenolate mofetil, and hydroxychloroquine were introduced, either as monotherapy or in combination. A smaller subgroup with refractory disease or severe organ involvement required biologic therapies, mainly infliximab, occasionally combined with methotrexate.

Within this heterogeneous therapeutic framework, our findings do not support modifying sarcoidosis-directed treatment based solely on the presence of migraine [7]. Migraine should be regarded as a comorbid primary headache disorder, rather than a direct therapeutic target of sarcoidosis treatment [17]. Notably, systemic therapies commonly used in sarcoidosis may variably influence headache patterns: corticosteroids may transiently improve migraine symptoms through anti-inflammatory effects, but may also exacerbate headache frequency or severity during tapering or prolonged use [18]. Conversely, immunosuppressive agents are not expected to directly modulate migraine pathophysiology.

The association between migraine and reduced DLCO suggests that patients with sarcoidosis and migraine may represent a subgroup with greater overall disease complexity, potentially warranting closer clinical monitoring rather than therapeutic escalation [7]. From a practical standpoint, accurate recognition of migraine is crucial to avoid unnecessary intensification of immunosuppressive therapy and to ensure appropriate referral to migraine specialists for guideline-based management [19].

### 4.2. Limitations of the Study

Several limitations must be acknowledged. The retrospective design, limited sample size, and potential for residual confounding restrict the ability to draw causal inferences. In addition, the diagnosis of migraine was based on clinical records rather than on a standardized, prospective headache assessment, which may have introduced misclassification bias or underreporting of headache phenotypes. The lack of detailed information on headache characteristics, including frequency, severity, duration, and response to treatment, further limits the granularity of the analysis.

Moreover, several clinically relevant variables, including inflammatory markers (e.g., CRP), were only partially available and characterized by a high proportion of missing data, as detailed in Supplementary Table S1. Other important factors, such as IL-6 levels, corticosteroid dose and duration, fatigue, sleep disorders, and autonomic dysfunction, were not systematically collected due to the retrospective design and therefore could not be included in the multivariable analysis. Missing data appeared to be broadly randomly distributed and mainly reflected the non-systematic availability of specific tests in routine clinical practice, rather than identifiable patient- or disease-related factors.

As a result, residual confounding cannot be excluded, and the observed association between migraine and DLCO may be partially influenced by unmeasured clinical variables.

Although the inclusion of patients from multiple centers may enhance the external validity of the findings, it may also introduce variability in clinical practice, diagnostic approaches, and data collection methods, potentially affecting data consistency. Furthermore, the inherent heterogeneity of sarcoidosis, together with differences in referral patterns across centers, may have influenced patient selection and clinical characteristics. Additional unmeasured factors, including psychosocial variables, may also have contributed to both migraine occurrence and pulmonary function outcomes.

In this context, the observed association between migraine and DLCO should be interpreted with caution and considered exploratory and hypothesis-generating, as the study design does not allow causal inference. While potential biological mechanisms can be hypothesized, they remain speculative and should be clearly distinguished from evidence-based findings.

## 5. Conclusions

In conclusion, migraine emerges as a frequent and clinically relevant comorbidity in sarcoidosis, and its association with lower DLCO values suggests a potential link with a greater functional disease burden. Although the biological basis of this relationship remains to be fully elucidated, it is plausible that shared inflammatory, vascular, and neuroimmune pathways may contribute to both pulmonary dysfunction and headache susceptibility. In this context, migraine should not be overlooked as a secondary manifestation of sarcoidosis, but rather recognized as a coexisting condition that may independently and synergistically impact patient-reported outcomes, functional status, and overall quality of life.

These findings highlight the importance of a multidisciplinary and patient-centered approach, integrating pulmonology, neurology, and headache expertise to improve diagnostic accuracy and optimize therapeutic strategies. A more systematic assessment of headache disorders in patients with sarcoidosis may help reduce misdiagnosis, avoid unnecessary investigations, and ensure appropriate, targeted management.

Future research should focus on large, prospective, multicenter studies with standardized phenotyping of both sarcoidosis and headache disorders, including the use of validated headache-specific instruments, such as the Migraine Disability Assessment (MIDAS) and the Headache Impact Test (HIT-6), in order to allow a more comprehensive assessment of headache burden and more refined phenotype–outcome correlations. In sarcoidosis, the integration of advanced analytical approaches, including AI and machine learning, together with the use of structured and validated radiologic classifications, may enable the identification of distinct clinical phenotypes and predictive models of disease burden. Coupled with precision medicine strategies, such approaches could facilitate the development of tailored interventions based on individual clinical, functional, and biomarker profiles, ultimately improving outcomes in this complex and heterogeneous patient population.

## Figures and Tables

**Table 1:** Clinical characteristics, comorbidities and respiratory function of patients (N = 72).

Variable	N (%)
Family history of sarcoidosis	
Yes	2 (2.8)
No	70 (97.2)
Risk factors (smoke and/or environmental/occupational exposure)	
Present	27 (37.5)
Absent	39 (54.2)
Not reported	6 (8.3)
BMI	
Underweight	1 (1.4)
Normal weight	22 (30.5)
Overweight	19 (26.4)
Grade I obesity	8 (11.1)
Grade II obesity	3 (4.2)
Grade III obesity	1 (1.4)
Not reported	18 (25)
Migraine	
Yes	21 (29.2)
No	51 (70.8)
Comorbidities	
Pleuro-pulmonary	11 (15.3)
Dyslipidemia	9 (12.5)
Hypertension	22 (30.6)
Diabetes	4 (5.6)
Atherosclerosis	5 (6.9)
Abnormal respiratory function	
Yes	28 (38.9)
No	32 (44.4)
Not reported	12 (16.7)
FEV_1_ (% predicted)	
≥80% (normal)	32 (44.4)
70–79% (mild)	10 (13.9)
60–69% (moderate)	2 (2.8)
50–59% (moderate-to-severe)	3 (4.2)
<50% (severe)	7 (9.7)
<30% (very severe)	3 (4.2)
Not reported	15 (20.8)
FVC (% predicted)	
≥80% (normal)	52 (72.2)
<70% (reduced)	5 (6.9)
Not reported	15 (20.8)
DLCO	
Normal	21 (29.2)
Slightly reduced	25 (34.7)
Moderate reduction	8 (11.1)
Severely reduced	2 (2.8)
Not reported	16 (22.2)
TLC (% predicted)	
80–120% (normal)	32 (44.4)
70–79% (slightly reduced)	11 (15.3)
>120% (hyperinflation)	2 (2.8)
Not reported	27 (37.5)
Therapy	
Steroids only	19 (26.39)
Steroids, immunosuppressants, immunomodulators, biologics	4 (5.55)
Steroids, immunosuppressants, immunomodulators	4 (5.55)
Steroids, immunomodulators	6 (8.34)
Steroids, immunosuppressants	5 (6.94)
Immunosuppressants only	5 (6.94)
Immunomodulators only	1 (1.39)
Biologics only	1 (1.39)
Nothing	15 (20.84)
Not reported	12 (16.67)

**Table 2:** Multivariable linear regression model evaluating the association between migraine and DLCO, adjusted for age, sex, BMI, and major comorbidities. Migraine onset was significantly associated with lower DLCO values, whereas all other variables were not statistically significant (95% CI including zero). Model statistics: R^2^ = 0.25; F(10,44) = 1.50; *p* = 0.17.

Variable	β Coefficient	Standard Error	95% CI	*p*-Value
Intercept	73.40	23.91	25.21 to 121.60	—
Age	−0.22	0.30	−0.81 to 0.38	NS
Sex	−6.80	5.75	−18.39 to 4.80	NS
Migraine onset	−19.15	7.01	−33.28 to −5.02	<0.05
Family history	2.49	3.82	−5.21 to 10.20	NS
Pleuro-pulmonary diseases	5.64	5.82	−6.09 to 17.37	NS
BMI	0.78	0.64	−0.52 to 2.08	NS
Dyslipidemia	−7.12	6.74	−20.71 to 6.48	NS
Hypertension	0.30	7.52	−14.85 to 15.46	NS
Diabetes	11.04	6.08	−1.22 to 23.31	NS
Atherosclerosis	−8.04	9.24	−26.66 to 10.59	NS

## Data Availability

Full anonymous data from the survey are available on request.

## Supplementary Materials

The following supporting information can be downloaded at https://www.mdpi.com/article/10.3390/life16050762/s1: Table S1: Laboratory parameters of the study population. Continuous variables are reported as mean ± standard deviation. For each parameter, the number and percentage of patients with available data and missing values are provided. BUN = Blood Urea Nitrogen, ESR = Erythrocyte Sedimentation Rate, CRP = C-reactive Protein.
