# Supplementary material for: OUtcome and Clinical Characteristics of Primary Headache in Patients with Sarcoidosis: The OUCH! Study
Source: Life (Basel). 2026 May 2;16(5):762. doi: 10.3390/life16050762 (PMC13208288; doi:10.3390/life16050762)
Supplement: Supplementary file 1 [file life-16-00762-s001.zip › life-4254013-supplementary.pdf]

Supplementary Table S1. Laboratory parameters of the study population. Continuous variables are reported as mean ± standard deviation. For each parameter, the number and percentage of patients with available data and missing values are provided. BUN = Blood Urea Nitrogen, ESR = Erythrocyte Sedimentation Rate, CRP = C-reactive Protein.

Laboratory parameters	Mean + Standard Deviation	N (%)	Not reported N (%)
Hemoglobin (g/dL)	14.02 ± 1.59	28 (38.89)	44 (61.11)
Creatinin (mg/dL)	1.53 ± 1.98	27 (37.5)	45 (62.5)
BUN (mg/dL)	32.1 ± 7.45	4 (5.56)	68 (94.44)
Colesterol (mg/dL)	181.63 ± 32.11	13 (18.06)	59 (81.94)
LDL (mg/dL)	136.46 ± 63.65	10 (13.89)	62 (86.11)
HDL (mg/dL)	48.95 ± 9.61	12 (16.66)	60 (83.34)
Triglycerides (mg/dL)	98.61 ± 58.20	12 (16.66)	60 (83.34)
Sodium (mmol/L)	140.23 ± 2.68	13 (18.06)	59 (81.94)
Potassium (mmol/L)	4.16 ± 0.55	13 (18.06)	59 (81.94)
Calcium (mmol/L)	8.45 ± 2.42	23 (31.95)	49 (68.05)
ESR	12.75 ± 11.93	20 (27.77)	52 (72.23)
CPR	4.77 ± 9.3	23 (31.95)	49 (68.05)
